# Heterogeneous Intermediate Phenotypes of Cancer Cells with Varying Ki-67-Positivity Rates, Including Histologically HCC-like and NEC-like Cells, in Liver MiNEN

**DOI:** 10.3390/ijms27083390

**Published:** 2026-04-09

**Authors:** Sumie Ohni, Yoko Nakanishi, Yukari Hirotani, Ryosuke Toyonaka, Osamu Aramaki, Yukiyasu Okamura, Shinobu Masuda, Makoto Makishima, Mariko Esumi

**Affiliations:** 1 Division of Oncologic Pathology, Department of Pathology and Microbiology, Nihon University School of Medicine, Tokyo 173-8610, Japan; ohni.sumie@nihon-u.ac.jp (S.O.); nakanishi.youko@nihon-u.ac.jp (Y.N.); obana.yukari@nihon-u.ac.jp (Y.H.); masuda.shinobu@nihon-u.ac.jp (S.M.); 2 Division of Digestive Surgery, Department of Surgery, Nihon University School of Medicine, Tokyo 173-8610, Japan; toyonaka.ryosuke@nihon-u.ac.jp (R.T.); aramaki.osamu@nihon-u.ac.jp (O.A.); okamura.yukiyasu@nihon-u.ac.jp (Y.O.); 3 Division of Biochemistry, Department of Biomedical Sciences, Nihon University School of Medicine, Tokyo 173-8610, Japan

**Keywords:** cancer cell heterogeneity, mixed neuroendocrine–non-neuroendocrine neoplasm, intermediate phenotype, Ki-67, TERT, p53, mixed hepatocellular carcinoma–neuroendocrine carcinoma, transdifferentiation

## Abstract

Mixed hepatocellular carcinoma (HCC)–neuroendocrine carcinoma (NEC) is a major type of liver mixed neuroendocrine–non-neuroendocrine neoplasm (MiNEN). Primary liver NEC, which is very rare, is mostly associated with HCC rather than pure NEC. To characterize the cancer cell heterogeneity of the HCC and NEC components, we comprehensively analyzed the protein expression of three cancer cell biological markers (TERT, Ki-67, and p53) and five differentiation markers (one hepatocyte marker and four neuroendocrine markers) via immunohistochemistry and immunofluorescence using curative resection tissues from three patients with liver MiNEN. TERT/Ki-67/p53 proteins, which are related to cell proliferation and malignancy, were independently expressed in the HCC and NEC components; Ki-67 was highly expressed among the three proteins in both cancer components, and the expression of all three markers was higher in the NEC component than in the HCC component. Despite the intracomponent and intercomponent heterogeneity, the expression signatures of the three markers were similar between the two components, potentially suggesting a common origin of mixed HCC-NEC. An in-depth exploration of intracomponent heterogeneity using differentiation markers revealed multiple intermediate phenotypes of cancer cells, i.e., HCC-like and NEC-like cells, mainly in the HCC component. Histologically NEC-like cells rather than HCC-like cells tended to have an intermediate percentage of Ki-67-positive cells, compared with NEC cells. The spatial distribution of various intermediate cancer cell phenotypes suggests that mixed HCC-NEC may involve the transdifferentiation from HCC cells to NEC cells through the dedifferentiation of HCC.

## 1. Introduction

Mixed neuroendocrine–non-neuroendocrine neoplasms (MiNENs) were first described in the 2019 World Health Organization (WHO) Classification of Neoplasms of the Digestive System. MiNENs are mixed neoplasms with substantial neuroendocrine and non-neuroendocrine components (>30% of either one) [1,2]. Liver MiNEN, which accounts for 0.5% of primary liver tumors, appears to be more common than pure neuroendocrine carcinoma (NEC) and has two subtypes, mixed hepatocellular carcinoma (HCC)-NEC and mixed cholangiocarcinoma–NEC, with more cases of the former than the latter (see Box 1.01 in WHO 2019) [1]. Compared with HCC, hepatic MiNEN is highly aggressive and is associated with poor prognosis. Therefore, differentiation from NEC cells to HCC cells is unlikely. The simultaneous development of HCC cells and NEC cells or differentiation from HCC cells to NEC cells is also possible considering the incidence rates of HCC and pure NEC in the liver [2]. Although there are clinical case reports of mixed HCC-NEC [3,4], comprehensive studies on the mechanisms underlying its occurrence and progression are still rare.

In this study, we explored in detail the diversity of cancer cells in hepatic MiNEN tissue to evaluate cancer development and progression. To characterize cancer cell features, we examined the protein expression of three cancer cell biological markers and five cancer cell differentiation markers by immunohistochemistry (IHC) and immunofluorescence (IF). Two cancer cell biological markers, telomerase reverse transcriptase (TERT) and p53, are frequently overexpressed in HCC and are associated with driver mutations [5,6]. Another cancer cell biological marker, Ki-67, is a popular cancer proliferation marker; a high Ki-67-positivity rate is associated with poor prognosis in patients with HCC [7], and the Ki-67-positivity rate is very high in liver NEC [2]. Using these three markers, we previously clarified the quantitative phenotypic heterogeneity of cancer components and intracomponent microheterogeneity in combined HCC–cholangiocarcinoma (CC) by IHC [5]. In this study, using three cases of MiNEN, we comprehensively analyzed the expression of three cancer cell markers and five cancer cell differentiation markers, one hepatocyte (HepPar1, hepatocyte paraffin 1) and four neuroendocrine markers (CGA, chromogranin A; SYN, synaptophysin; CD56, neural cell adhesion molecule; INSM1, insulinoma-associated protein 1), in MiNEN tissues as a whole; interestingly, we found certain cancer cell phenotypes (i.e., HCC-like and NEC-like cells) in the HCC component, indicating the presence of intermediate cancer cell phenotypes in MiNEN.

## 2. Results

### 2.1. Immunohistochemistry (IHC) of Differentiation Markers in Each Cancer Component of Mixed Hepatocellular Carcinoma (HCC)–Neuroendocrine Carcinoma (NEC)

**Figure 1:**
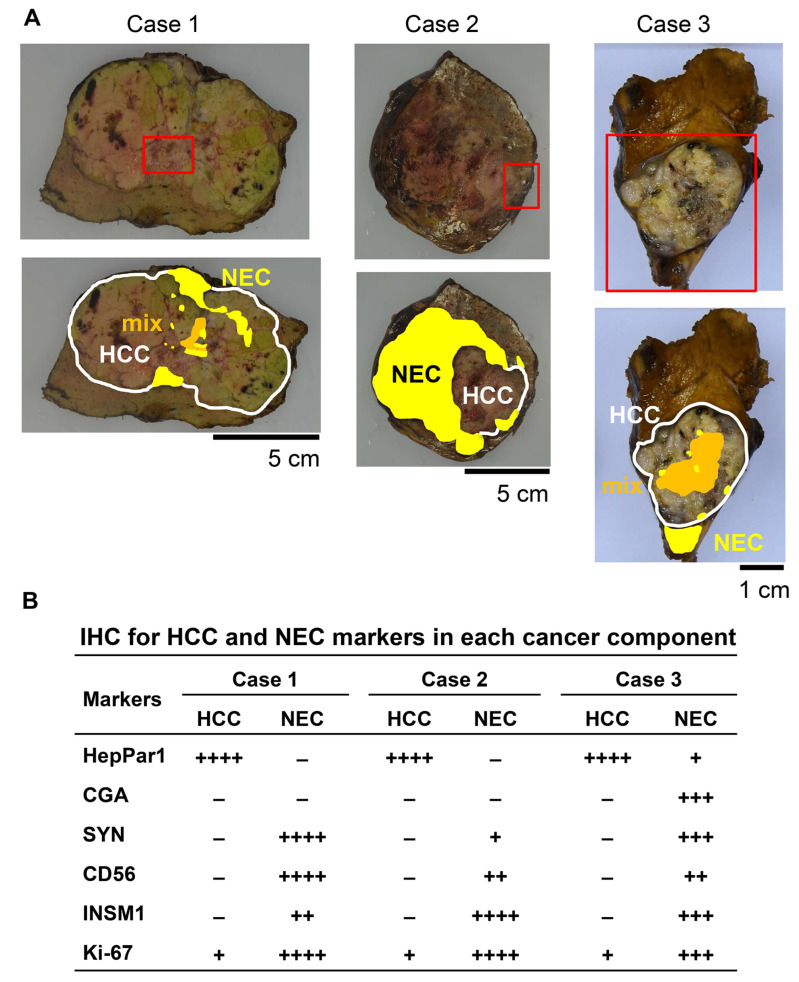
Three cases of mixed hepatocellular carcinoma (HCC)–neuroendocrine carcinoma (NEC). (**A**) Representative gross cross-section of a curatively resected liver. Histological macrofeatures of HCC (white) and NEC (yellow) components, including mixed HCC-NEC components (orange), were visualized by hematoxylin and eosin (H&E) staining of thin sections for all areas of the cross-section (lower panels). The red square indicates the histological specimen used for this study (upper panels). (**B**) Summary of IHC for HCC and NEC markers and Ki-67 in each cancer component. HCC and NEC were histologically characterized by H&E staining. The populations of positive cells are shown as ++++ (>80%), +++ (50 to 80%), ++ (15 to 50%), + (<15%) and – (0%). HepPar1, hepatocyte paraffin 1; CGA, chromogranin A; SYN, synaptophysin; CD56, neural cell adhesion molecule; INSM1, insulinoma-associated protein 1.

Partial livers from three patients who underwent curative liver tumor resection (Table S1) contained nodular-type and milky white tumors with marked hemorrhagic necrosis (Figure 1A). The tumor specimens were histologically diagnosed as mixed HCC-NEC using IHC for HCC and NEC markers (Figure S1), and the distribution of HCC, NEC and a mixture of both components varied across the three cases (Figure 1A). When the population of cells positive for these markers was semiquantitatively measured in each cancer component, the HCC component was strongly positive for hepatocyte paraffin 1 (HepPar1) in all three cases, but the NEC component was differentially positive for four NEC markers (Figure 1B and Figure S1). Ki-67 expression was highly positive in the NEC component but not in the HCC component in all three cases (Figure 1B and Figure S1). We quantitatively measured the number of Ki-67-positive cells in each cancer component and compared the findings, which are described below.

### 2.2. Protein Expression of Telomerase Reverse Transcriptase (TERT), Ki-67, and p53 in Multiple Components of Mixed HCC-NEC

**Figure 2:**
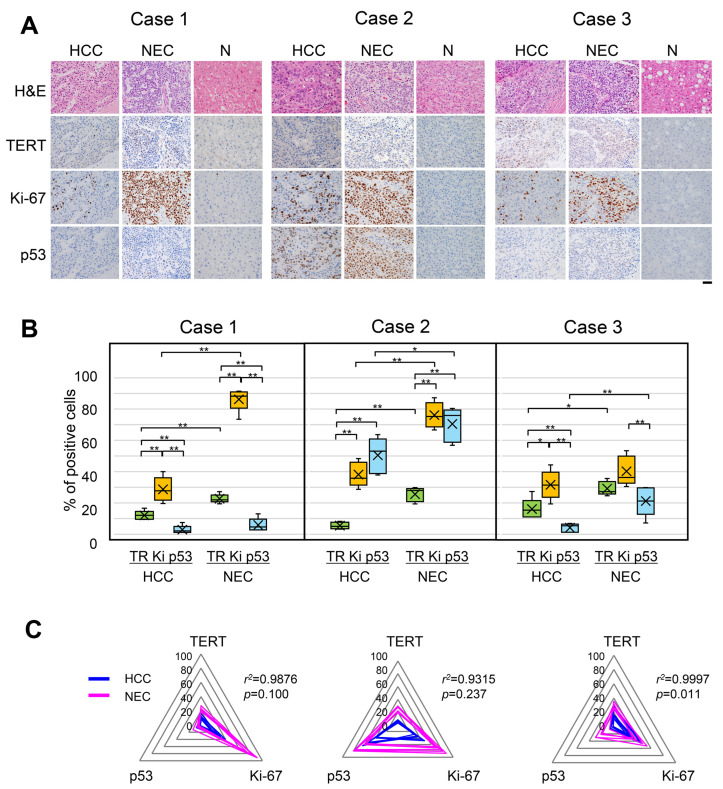
Immunohistochemistry (IHC) images of TERT, Ki-67 and p53 protein expression in the HCC and NEC components and nontumorous liver (N) region of mixed HCC-NEC. (**A**) Representative images of H&E staining and IHC for TERT, Ki-67 and p53 in serial thin sections. The bars indicate 50 µm. H&E, hematoxylin and eosin; TERT, telomerase reverse transcriptase; HCC, hepatocellular carcinoma; NEC, neuroendocrine carcinoma. (**B**) Percentages of cells positive for TERT, Ki-67 and p53 in each cancer component. The values were obtained from the same field of view for serial thin sections and represent five fields of view for each component. TR, TERT; Ki, Ki-67. **, *p* < 0.01; *, *p* < 0.05 (Mann–Whitney U test). (**C**) Radar charts of the positivity rate of three markers in five fields of view for each cancer component: HCC (blue) and NEC (magenta). Linear regression of the positivity rate of three markers between the two cancer components was analyzed by ordinary least squares estimation.

In addition to Ki-67, the protein expression of the cancer-related markers TERT and p53 in serial thin sections was assessed by IHC to examine the relationships among these three markers at the intercomponent and intracomponent levels. Like in our previous study on combined HCC-CC, in which both the HCC and CC components had the same *TERT* promoter mutation, known as a driver mutation of HCC [5], both cancer components of all three mixed HCC-NEC cases were positive for TERT protein expression in the present study; however, the nontumorous region was mostly negative (Figure 2A and Figure S2A). In the same fields of serial thin sections that were positive for TERT, the nuclei of cancer cells were positive for Ki-67 and p53 proteins, but the three markers were expressed independently of each other and to different degrees (Figure 2A,B). Among the three markers, Ki-67 was highly expressed in both the HCC and NEC components, except for case 2; in case 2, p53 expression occurred to a degree similar to that of Ki-67 in both cancer components (Figure 2B). All three markers were more highly expressed in the NEC component than in the HCC component. The distribution of cancer cells positive for the three markers was random but neither mutually exclusive nor inclusive. Thus, the three markers were independently expressed in the HCC and NEC components, suggesting that intracomponent cancer cells were phenotypically heterogeneous.

However, this phenotypic heterogeneity was quite similar between the two cancer components (Figure 2C and Figure S2B,C). The expression profiles of the three markers were compared between the HCC and NEC components using radar charts, and the correlations were statistically analyzed. To confirm phenotypic heterogeneity and intercomponent similarity, we measured the maximum positive rate of the markers in each cancer component (Figure S2B) and detected a statistically strong correlation between the two cancer components individually in the three cases (Figure S2C). These results suggest that the HCC and NEC components were similar in origin in all three cases.

### 2.3. Comprehensive Mapping of Histopathology and Double Immunofluorescence (IF) for Hepatocyte Paraffin 1 (HepPar1) and Insulinoma-associated Protein 1 (INSM1)

**Figure 3:**
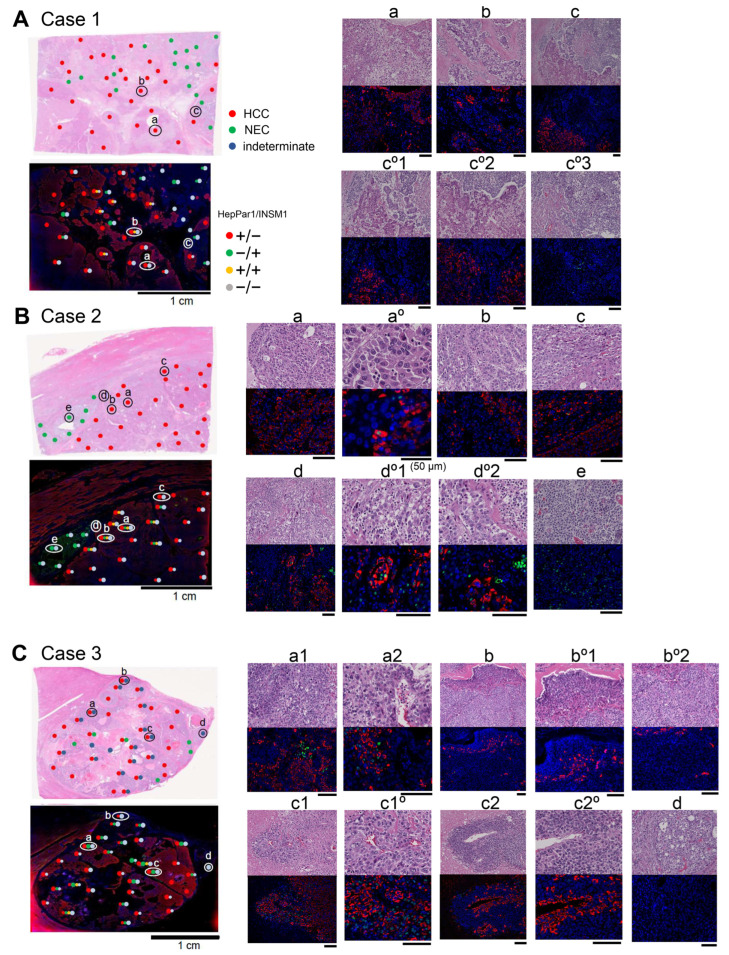
Double immunofluorescence (IF) for HepPar1 and INSM1 compared with histological images. Serial thin sections were stained with H&E (**upper**) and double IF (**lower**). Whole images of thin sections and microscopy-enhanced images of representative areas (a to e) are shown on the left and right, respectively. Mapping of histological diagnosis is shown in whole H&E images (red, HCC; green, NEC; dark blue, indeterminate) and that of HepPar1/INSM1 double IF staining is shown in whole IF images (red, +/−; green, −/+; yellow, +/+; gray, −/−). HepPar1, hepatocyte paraffin 1; INSM1, insulinoma-associated protein 1. Scale bars, 100 µm. (**A**) Case 1. a, b, HCC areas; c, borders of HCC and NEC. The images in c°1, c°2 and c°3 are high-magnification images of c. (**B**) Case 2.a, b, c, HCC areas; d, borders of HCC and NEC; e, NEC area. a°, d°1, and d°2 are magnified images of a, d, and d, respectively. The scale bars are 100 µm except for those marked 50 µm. (**C**) Case 3. a to c, indeterminate cell-containing areas with and without HepPar1 expression; d, indeterminate NEC-like cell area. a1 and a2, magnified images from area a; b°1 and b°2, magnified images from b; c1° and c2°, magnified images from c1 and c2, respectively.

For the definitive identification of HCC and NEC cells or indeterminate cancer cells, we comprehensively examined and compared the histopathology and double IF results for HepPar1 (hepatocyte marker) and INSM1 (neuroendocrine cell marker). In control IF staining of the HCC case, both tumor and nontumor regions were strongly and uniformly positive for HepPar1 but negative for INSM1 (Figure S3). The NEC components (Figure 3A(c°3),B(e)) were completely negative for HepPar1 but negative or positive, depending on the patient, for INSM1. In case 1, both cancer components were relatively clearly separated (Figure 3A, hematoxylin and eosin (H&E) image map). However, in the HCC component, HepPar1 staining was occasionally weak, patchy and negative (Figure 3A, IF image map, a-c). These atypical HCC cells exhibited histopathological changes in H&E images, such as clear cell changes and an increased nuclear–cytoplasmic ratio (Figure 3A(a)), as well as hyperchromatic nuclei and an increased cell density (Figure 3A(b,c°1,c°2)). HCC cells at the boundary of the NEC were heterogeneously atypical in terms of HepPar1 staining (patchy to negative) and histological morphology (HCC-like to NEC-like) (Figure 3A(c,c°1,c°2)). The NEC component was weakly positive for INSM1 (Figure 3A(c,c°1,c°3)). These HepPar1(−) HCC-like cells were also negative for other NEC markers, such as chromogranin A (CGA) and synaptophysin (SYN).

Additionally, in case 2, both cancer components were clearly separated (Figure 3B, H&E image map), but HepPar1(−) HCC cells were frequently observed (Figure 3B, IF image map). These atypical HCC cells (patchy positive or negative for HepPar1) were slightly morphologically altered and mostly exhibited negative staining but occasionally scattered positive staining for INSM1 (Figure 3B(a–d)). Infrequently, HepPar1/INSM1-double-positive cells were observed (Figure 3B(a°)), but these cells undeniably overlapped with individual single marker-positive cells. These HCC-like cells were quite different from NEC cells positive for INSM1 (Figure 3B(e)); intermingling of HepPar1(+) cells and INSM1(+) cells was observed in the atypical HCC region adjacent to the NEC component (Figure 3B(d,d°1,d°2)).

**Figure 4:**
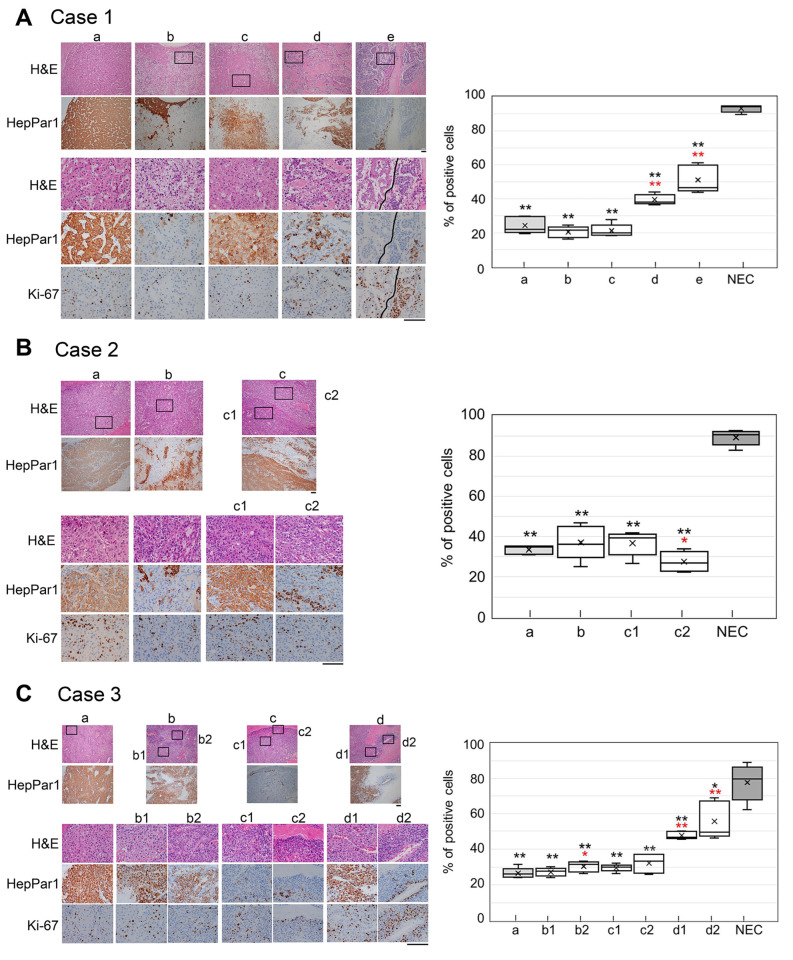

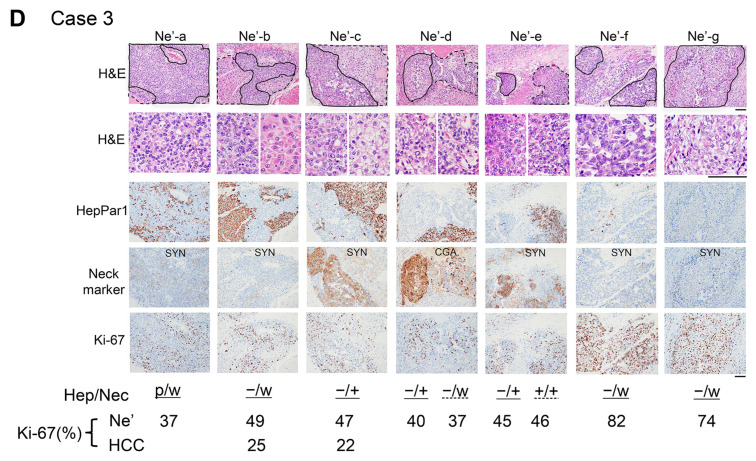
Ki-67-positivity rate in multiple areas containing atypical HCC-like and NEC-like cells. (**A**–**C**) Representative images of HCC control (a) and atypical staining (b to e) for HepPar1 and Ki-67 in tissue samples from cases 1 (**A**), 2 (**B**) and 3 (**C**) (**left**) and individual Ki-67-positivity rates represented with a box plot of measurements from five fields of view (**right**). The images in the squares in the upper two panels are magnified and are shown in the lower three panels. Scale bars, 100 µm. HCC-Ki-67 (a) and the maximum value of NEC-Ki-67 (NEC) shown in Figure S2 were used as controls for individual comparisons of b to e by the Mann–Whitney U test. ** (*p* < 0.01) and * (*p* < 0.05) are shown in black for the NEC comparison and in red for the HCC (a) comparison. (**D**) Representative H&E and IHC images of indeterminate NEC-like cells (Ne’) in the tissue sample from case 3 and the percentage of Ki-67-positive cells in the indicated area. The percentage of Ki-67-positive cells was determined by counting two or three fields of view in the pinpointed area (solid and dotted lines, respectively) and is presented as the average. Hep/Nec, IHC results for HepPar1 (Hep) and three NEC markers (Nec) (also shown in Figure S4); p, patchy; w, weakly positive. Scale bars, 100 µm. SYN, synaptophysin; CGA, chromogranin A.

In case 3, the HCC component had a large number of indeterminate cancer cells (Figure 3C, H&E image map). Like in cases 1 and 2, HepPar1(−)/INSM1(−) cells were observed as HCC-like cells (Figure 3C(a1 left,a2 right,b,c2)), and the HCC-like cells in the HepPar1-patchy area were intermingled with INSM1(+) cells (Figure 3C(a1,a2 left,c1)); it was difficult to morphologically discriminate these double-negative cells or INSM1(+) cells from HepPar1(+) cells. A histologically NEC-like component but not an HCC component was also observed and was unexpectedly negative for all NEC markers, including INSM1, as well as for HepPar1 (Figure 3C(d)), leading us to conclude that these cells were indeterminate. In addition, a variety of indeterminate NEC-like but not HCC-like cancer cells were observed in case 3 (Figure S4 and Figure 4D). These NEC-like cells were indistinguishable from the NEC cells and exhibited three staining patterns: HepPar1(−)/NEC marker(−), HepPar1(−)/NEC marker(+), and intermingling of HepPar1(+) and NEC marker(+) (Figure S4 and Figure 4D).

Thus, we found a variety of cancer cells that were histologically different from typical HCC and NEC cells in mixed HCC-NEC tissues. These atypical cancer cells also exhibited a variety of intermediate phenotypes according to marker expression: (1) HCC-like cells that were patchy positive or negative for HepPar1; (2) HCC-like cells that were patchy positive for HepPar1 and intermingled with NEC marker-positive ones; (3) NEC-like cells negative for both HepPar1 and NEC markers; and (4) NEC-like cells positive for NEC markers.

### 2.4. Ki-67-Positivity Rate in a Variety of Atypical HCC-like Cells and NEC-like Cells

**Figure 5:**
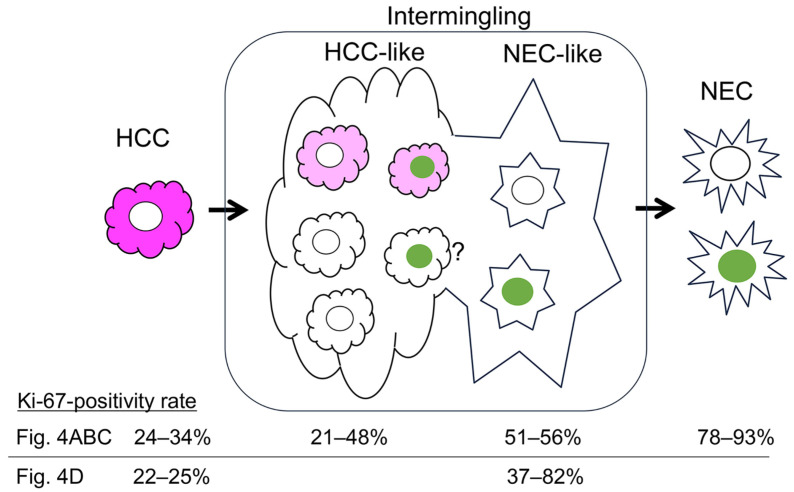
Schematic of transdifferentiation in mixed HCC-NEC based on multiphenotype HCC cells (HCC-like) and indeterminate NEC-like cells, together with the Ki-67-positivity rate. The HCC component contains numerous atypical HCC cells with HepPar1-patchy and -negative staining (middle-left). Occasionally, in the HepPar1-patchy area, HCC-like cells intermingle with HepPar1(−)/INSM1(+) cells, making it difficult to morphologically identify HCC-like cells; however, HepPar1(+)/INSM1(+) HCC-like cells are rarely observed (middle). The percentage of Ki-67-positive cells observed in this study is shown individually for cells with multiple phenotypes. A significant increase in the percentage of Ki-67-positive cells was observed among NEC-like cells (middle-right). Magenta, HepPar1 (HCC marker); green, INSM1 (NEC marker).

To characterize the intermediate subtypes of cancer cells, the frequency of Ki-67-positive cells was assessed and compared with that of typical HCC and NEC components. Representative atypical HCC-like cells that were patchy positive or negative for HepPar1 had Ki-67-positivity rates similar to those of typical HCC cells (Figure 4A–C), except for areas that were histologically NEC-like rather than HCC-like (Figure 4A(d,e),C(d1,d2)); the Ki-67-positivity rates of these cells were intermediate and significantly different from those of both typical HCC and NEC cells (Figure 4A,C). Moreover, we measured the Ki-67-positivity rate of a variety of NEC-like cells in pinpointed regions of case 3 (Figure S4). These cells had higher Ki-67-positivity rates than did HCC cells (22–25%) (37% to 82%), exhibiting values similar to those of intermediate and NEC cells (78–93%) (Figure 4D). These results demonstrate that among the four subtypes of intermediate cancer cells described above, NEC-like cells but not HCC-like cells had intermediate Ki-67-positivity rates (Figure 5). The area of HepPar1(+) cell and INSM1(+) cell intermingling observed in the atypical HCC component also revealed an intermediate percentage of Ki-67-positive cells (Figure 3C(c1) and Figure 4C(d1),D(e—right)). Interestingly, NEC-like cells that were mostly negative for NEC differentiation markers had a high Ki-67-positivity rate, corresponding to typical NEC cells (Figure 4D(f,g)). Thus, a high Ki-67-positivity rate is closely associated with the histological phenotype of NEC cells rather than with the expression of NEC differentiation markers.

## 3. Discussion

Herein, we report new findings on cancer cell heterogeneity observed in mixed HCC-NEC from two perspectives: cancer cell properties and cancer cell differentiation. First, in terms of the variations in the expression of TERT, Ki-67 and p53 in each cancer component, we demonstrated that cancer cells exhibit intracomponent phenotypic heterogeneity (Figure 2), which was similar in the HCC and NEC components in each case (Figure 2C and Figure S2C). Interestingly, these findings suggest the possibility that the two cancer components develop from a common origin.

The TERT protein seems to play a role in the early development of HCC because its promoter mutation is known as an early driver mutation of HCC. Markers of a poor prognosis, the proteins Ki67 and p53 showed independent expression at the cell level and no relationship with TERT-positive cells in both HCC and NEC components; a certain signaling network among these cancer-related marker events does not directly exist, like in our previous study on combined HCC-CC [5]. However, all three markers were more highly expressed in the NEC component than in the HCC component (Figure 2B). Thus, the NEC component has a more malignant phenotype than the HCC component. Interestingly, even in the HCC component, a special area with a Ki67-high/p53-high positivity rate was found to be HepPar1-patchy positive, suggesting that some atypical HCC cells with HepPar1-patchy staining have a more malignant phenotype than typical HCC cells. Thus, many more malignant cancer properties for the NEC component seem to be involved in early metastatic recurrence and poor prognosis, namely very early metastatic recurrence from 2 to 4 months after resection, and a survival period of less than 2 years despite treatment in this study (Table S1). There were no relationships among tumor size, recurrence, prognosis, subtype of HCC-NEC (component ratio, component distribution such as combined, collision or mixture) or etiology using 23 cases from the literature review [3,8] and our study. Further comprehensive study involving a larger number of cases is necessary to clarify these relationships.

A literature review on the two-component comparison of the Ki-67-positivity rate in mixed HCC-NEC yielded results similar to those of our study in that the Ki-67-positivity rate for the NEC component was markedly greater than that for the HCC component; however, only a few case reports on p53 and none on TERT have been published (Table 1) [3,8,9,10,11,12,13,14]. The extremely high expression rate of both HCC-p53 and NEC-p53 is probably due to p53 mutations, as described in HCC cases [6] and in a case of mixed HCC-NEC (Table 1) [3]. The overexpression of the p53 protein occurred in a case-dependent manner in mixed HCC-NEC (Figure 2 and Table 1). To determine clonality, future studies should explore whether p53 mutations are the same in both cancer components. TERT protein expression was shown for the first time in the HCC and NEC components of mixed HCC-NEC. It will be interesting and useful to determine the clonality of the two cancer components in terms of the presence of *TERT* promoter mutations, as described previously [5].

There are a few case reports on the clonal analysis of HCC and NEC components, and the results of these studies suggest that mixed HCC-NEC has the same molecular and genetic features as HCC; major HCC driver mutations were observed in both cancer types [3,9,15]. Recent developments in spatial multiomic analysis have enabled the phylogeographic mapping of cancer evolution, revealing that cancer progression trajectories are accompanied by microenvironmental alterations [16,17]. This integrated single-cell and spatial transcriptomics approach is useful for determining the treatment for patients with biphenotypic cancers such as mixed HCC-NEC as well as for determining the mechanism underlying cancer evolution heterogeneity, as shown in a cHCC-CC study [18].

Second, in terms of the expression profiles of differentiation markers and Ki-67 in the HCC and NEC components, we demonstrated for the first time that there are multiple intermediate phenotypes of cancer cells, namely HCC-like cells and NEC-like cells, and that histologically NEC-like cells have an intermediate Ki-67-positivity rate or up to that of NEC (Figure 4). There are a few case reports referring to an intermediate type of cancer cell in mixed HCC-NEC: a cancer cell that coexpressed both HCC and NEC markers and was intermingled in the transitional zone of the HCC and NEC components [12,19]. This cancer cell type corresponds to one of four intermediate phenotypes in our study, but double-positive cells were very rare in the area of intermingling (Figure 3B,C). Shi C, et al. [9] also reported an atypical HCC component that was patchy positive for HepPar1 with focal expression of an NEC marker, findings that were similar to those observed in our study (Figure 3B,C and Figure 4D).

On the basis of our overall examination of mixed HCC-NEC tissues, the similarity of cancer biological phenotypes between HCC and NEC components and the spatial distribution of heterogeneous intermediate differentiation phenotypes, we propose that mixed HCC-NEC develops from a common origin and from the potential transdifferentiation of HCC cells to NEC cells through the dedifferentiation of HCC cells (Figure 5). However, genetic clonal analysis such as mutation analysis remains to be done to conclude a common origin of mixed HCC-NEC. On the other hand, it remains possible that latent cancer stem cells or hepatic progenitor cells bidirectionally differentiate into HCC and NEC components simultaneously. The major background liver disease of mixed HCC-NEC is viral and non-viral chronic hepatitis, similar to that of HCC, but liver cirrhosis was observed in only 38% of patients from the literature review [4] and in none of the three patients from our study (F1,F2,F1 in Table S1). Considering that mixed HCC-NEC develops more frequently from non-cirrhotic liver than cirrhotic liver, hepatic progenitor cell-related carcinogenesis, if any, is less involved in the development of mixed HCC-NEC because hepatic progenitor cells are activated under a highly fibrotic lesion [20].

Recently, cancer plasticity was shown to promote the development of MiNENs, and a sophisticated study on patient organoids with mixed small-cell NEC–adenocarcinoma of the uterine cervix involving the tracking of single cells revealed the existence of cells that undergo bidirectional differentiation toward NEC and adenocarcinoma [21]. Anticancer drug treatment has been shown to induce changes in differentiation at the single-cell level, suggesting the plasticity of cancer cells in MiNENs [21]. Recently, Irland, AS et al. reported that basal cells drive small-cell lung cancer plasticity, generating heterogeneity in tumors of all subtypes [22]. Plasticity itself is a target for cancer therapeutics. Thus, to elucidate the involvement of dedifferentiated cells or stem-like cells in hepatic MiNENs, single-cell and spatial multiomic analysis will be useful for determining the role of such cells in cancer progression.

## 4. Materials and Methods

### 4.1. Patients and Liver Specimens

Table S1 provides a summary of three patients with mixed HCC-NEC. Preoperatively, the tumors were positive for the following markers: protein induced by vitamin K absence or antagonist-II (PIVKA-II) in patient 1 and both PIVKA-II and alpha-fetoprotein (AFP) in patients 2 and 3. Therefore, the clinical diagnosis for these three patients was HCC. The tumor size was larger than 10 cm in patients 1 and 2 but less than 5 cm in patient 3. Recurrence occurred early, from 2 to 4 months after resection, and the survival period was less than 2 years despite treatment. Histologically, the three tumors were diagnosed as mixed HCC-NEC; the HCC component displayed trabecular growth and was well to moderately differentiated (Edmondson and Steiner grade 1 to 2) [23]. The NEC component displayed nest and cord growth with coarsely clumped chromatin. The fibrosis score of the nontumorous region ranged from F1 to F2 [24] (Table S1 and Figure S1). This study was approved by the Ethics Committee of Nihon University School of Medicine (approval no. RK-230912-4). Informed consent was obtained from the patients prior to the start of the study.

### 4.2. IHC

Serial thin sections (4 μm) of formalin-fixed, paraffin-embedded (FFPE) tissues were subjected to IHC for HepPar1, CGA, SYN, neural cell adhesion molecule 1 (NCAM1, CD56), INSM1, TERT, Ki-67 and p53. As shown in Table S2, thin FFPE sections were incubated with a primary antibody for 45 min at room temperature after antigen retrieval (for the anti-TERT antibody, the sections were incubated for 60 min). Afterward, the sections were incubated with Histofine Simple Stain MAX PO (MULTI; Nichirei Biosciences, Tokyo, Japan) as the secondary antibody for 30 min at room temperature; Envision+/HRP (Agilent Technologies, Santa Clara, CA, USA) was used as the secondary antibody for TERT. IHC staining was performed with 3,3′ diaminobenzidine chromogen (Wako Pure Chemical Industries, Osaka, Japan), and the sections were counterstained with hematoxylin. Images were captured using a system of Olympus BX41 Stereo Microscope, Olympus DP74 camera and cellSens Standard 4.1 software (Evident Scientific, Tokyo, Japan).

Using IHC for TERT, Ki-67 and p53, the proportion of antigen-positive cells was determined in each cancer component and compared as described previously [5]. Profiles of the positivity rates for TERT, Ki-67 and p53 were compared between the HCC and NEC components using a radar chart, and their similarity was estimated through linear regression of the three rates between the two components in each case analyzed via ordinary least squares estimation. Simple comparisons of the positivity rates of three markers in each cancer component were performed using the Kruskal–Wallis test with Bonferroni correction. A *p* value < 0.0166 was considered to indicate statistical significance.

### 4.3. IF Double Staining for HepPar1 and INSM1

Double IF was performed using the Opal multiplex IHC system (Akoya Biosciences, Marlborough, MA, USA), essentially following the manufacturer’s instructions. Thin sections were subjected to antigen retrieval using EnVision FLEX Target Retrieval Solution (pH 9.0) (Agilent Technologies) for 30 min at 95–99 °C and incubated with a primary antibody against HepPar1 (1:2000) for 30 min at room temperature and with a secondary antibody, i.e., Histofine Simple Stain MAX-PO (Nichirei Biosciences), for 30 min at room temperature. After signal amplification by incubation with Opal 620 Fluorophore (1:200, Akoya Biosciences) for 10 min, antibody stripping was performed by boiling the sections in AR6 buffer (pH 6, Akoya Biosciences) for 20 min. In the second cycle, the sections were similarly treated as in the first cycle but were incubated with a primary antibody against INSM1 (1:1000) for 30 min and then with Opal 520 Fluorophore (1:200, Akoya Biosciences) for 10 min. Then, the sections were counterstained with Spectral DAPI (4′,6-diamidino-2-phenylindole) (Akoya Biosciences) and mounted with Prolong Diamond Antifade Mountant (Thermo Fisher Scientific, Waltham, MA, USA). IF images were acquired using a BZ-X800L fluorescence microscope with BZ-X Viewer and BZ-X800 Analyzer software (1.1.30.19) (Keyence, Osaka, Japan).

### 4.4. Statistical Analysis

Statistical analysis was performed using the Mann–Whitney U test for the positivity rates of TERT, Ki-67 and p53 expression, as visualized via IHC, in the HCC, NEC and N samples. The positivity rate of the three markers was also analyzed using the Kruskal–Wallis test. Linear regression of the positivity rates of TERT, Ki-67, and p53 expression between the HCC component and the NEC component in each case was performed by ordinary least squares estimation. The positivity rate of Ki-67 in atypical HCC components was analyzed using the Mann–Whitney U test.

## Figures and Tables

**Table 1:** Literature review on Ki-67 and p53-positivity rates in cancer components from mixed HCC-NEC and clinical features.

Ref. Year [No.]	Age	Sex	Etiology	Ki-67 (%)		p53 (%)		Recurrence/Metastasis		Death Mo.	Alive Mo.
				HCC	NEC	HCC	NEC	Mo.	Tissue		
Our study	71	M	NBNC	30	93	6	12	4	Liver, spine	13	
	73	M	C	48	89	80	93	2	Adrenal gland, peritoneum, spleen, LNs	17	
	77	F	NBNC	44	78	19	49	3	Liver, peritoneum, LNs	6	
2025 [8]	73	M	NBNC	40–50	80			5	Lung, LNs, adrenal gland		14
2024 [3]	58	M	B	8	80		~70 *				9
2021 [10]	84	F	NBNC **	10	80						9
2021 [9]	64	F	C	40	75				Not mentioned		9
2020 [11]	50	M	C	10	70			13	Liver, LNs, bone	33	
2017 [12]	71	M	C	15 ***	59 ***				Liver	9	
	71	M	C						Liver	3	
	58	M	B								19
	50	M	B								19
	63	M	C								24
2006 [13]	50	M	C	10–20	70–80			4	Liver, mesentery, LNs		15
2004 [14]	71	M	C	27	51	14	37	5	Pelvic bone (NEC)		5

* Not mentioned, but calculated from Figure 3B–D of Reference [3] where p53 mutation was detected. ** No hepatitis. *** Average of 5 cases. HCC, hepatocellular carcinoma; NEC, neuroendocrine carcinoma; mo., month; M, male; F, female; B, hepatitis B; C, hepatitis C; NBNC, non-B, non-C hepatitis; LNs, lymph nodes.

## Data Availability

The original contributions presented in this study are included in the article/Supplementary Materials. Further inquiries can be directed to the corresponding authors.

## Supplementary Materials

The following supporting information can be downloaded at https://www.mdpi.com/article/10.3390/ijms27083390/s1.

## Abbreviations

The following abbreviations are used in this manuscript:

MiNEN	Mixed neuroendocrine–non-neuroendocrine neoplasm
WHO	World Health Organization
HCC	Hepatocellular carcinoma
NEC	Neuroendocrine carcinoma
CC	Cholangiocarcinoma
IF	Immunofluorescence
IHC	Immunohistochemistry
H&E	Hematoxylin and eosin
TERT	Telomerase reverse transcriptase
HepPar1	Hepatocyte paraffin 1
INSM1	Insulinoma-associated protein 1
CGA	Chromogranin A
SYN	Synaptophysin
NCAM1	Neural cell adhesion molecule 1
PIVKA-II	Protein induced by vitamin K absence or antagonist-II
AFP	Alpha-fetoprotein
FFPE	Formalin-fixed paraffin-embedded
DAPI	4′,6-Diamidino-2-phenylindole
