# Supplementary material for: Heterogeneous Intermediate Phenotypes of Cancer Cells with Varying Ki-67-Positivity Rates, Including Histologically HCC-like and NEC-like Cells, in Liver MiNEN
Source: Int J Mol Sci. 2026 Apr 9;27(8):3390. doi: 10.3390/ijms27083390 (PMC13116802; doi:10.3390/ijms27083390)
Supplement: Supplementary file 1 [file ijms-27-03390-s001.zip › ijms-4152889-supplementary.pdf]

Table S1. Clinicopathological features of patients with mixed HCC-NEC at the time of curative resection.

Variable	Patient 1	Patient 2	Patient 3
Age	71	73	77
Sex	male	male	female
Tumor size (mm)	105x75	120x80	45x25
Nontumor histology*	A1F1 Steatosis	A1F2	A1F1 Steatosis
Recurrence (months after resection)	4	2	3
Survival (months after resection)	13	17	6
T-Bil (mg/dL)	0.44	0.95	0.51
AST (U/L)	79	46	20
ALT (U/L)	103	42	20
γ-GTP (U/L)	86	108	16
LDH (U/L)	192	383	177
ALP (U/L)	109	134	59
CEA (ng/mL)	2	3	5
CA19-9 (U/mL)	5	27	19
PIVKA-II (mAU/mL)	1280	14672	753
AFP (ng/mL)	20	1963	1676
Alcohol intake (g/day)	12	60	0
Underlying disease	DM		HT, DM, AS
Etiology	NBNC (MASLD)	C	NBNC (MASLD)

*The grades of necro-inflammatory activity (A) and fibrosis (F) refer to the new Inuyama classification of chronic hepatitis [24].

T-Bil, total bilirubin; AST, aspartate aminotransferase; ALT, alanine aminotransferase; γ-GTP, γ-glutamyl transpeptidase; LDH, lactate dehydrogenase; ALP, alkaline phosphatase; CEA, carcinoembryonic antigen; CA, carbohydrate antigen; PIVKA-II, protein induced by vitamin K antagonist-II; AFP, α-fetoprotein; DM, diabetes mellitus; HT, hypertension; AS, aortic stenosis; NBNC, non-B, non-C hepatitis; C, hepatitis C; MASLD, metabolic dysfunction-associated steatotic liver disease.

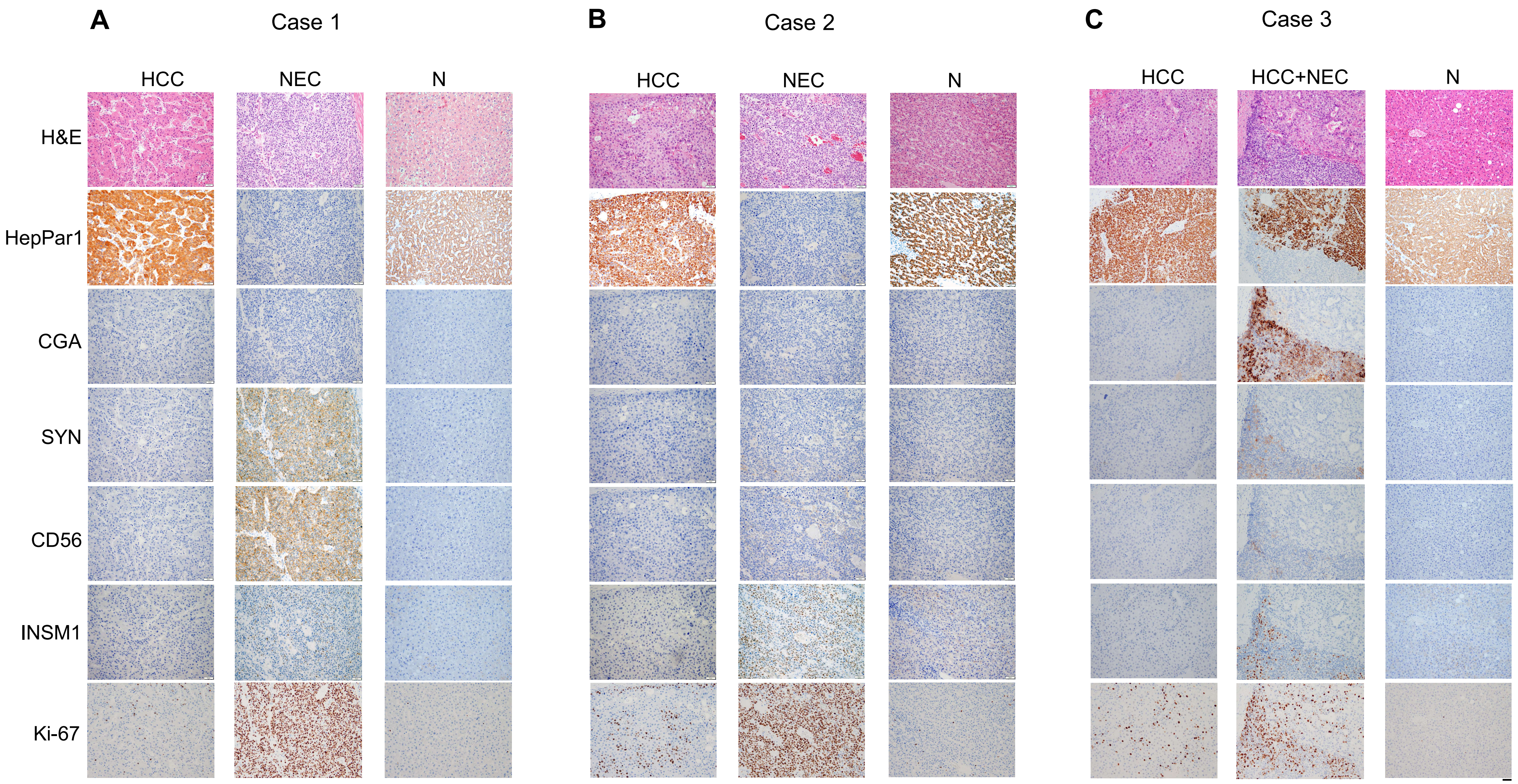

Figure S1. Representative images of three regions—the HCC component, the NEC component and a nontumor liver (N) region—after immunohistochemical staining for HepPar1, CGA, SYN, CD56, INSM1 and Ki-67. (A) Case 1. NEC was strongly positive for SYN, CD56 and INSM1 (nuclei). (B) Case 2. NEC was strongly positive for INSM1 (nuclei). (C) Case 3. NEC was positive for CGA (strong), SYN, CD56 and INSM1 (nuclei). HCC, hepatocellular carcinoma; NEC, neuroendocrine carcinoma; HepPar1, hepatocyte paraffin 1; CGA, chromogranin A; SYN, synaptophysin; CD56, NCAM1 (neural cell adhesion molecule 1); INSM1, insulinoma-associated protein 1. Bars, 50 μm.

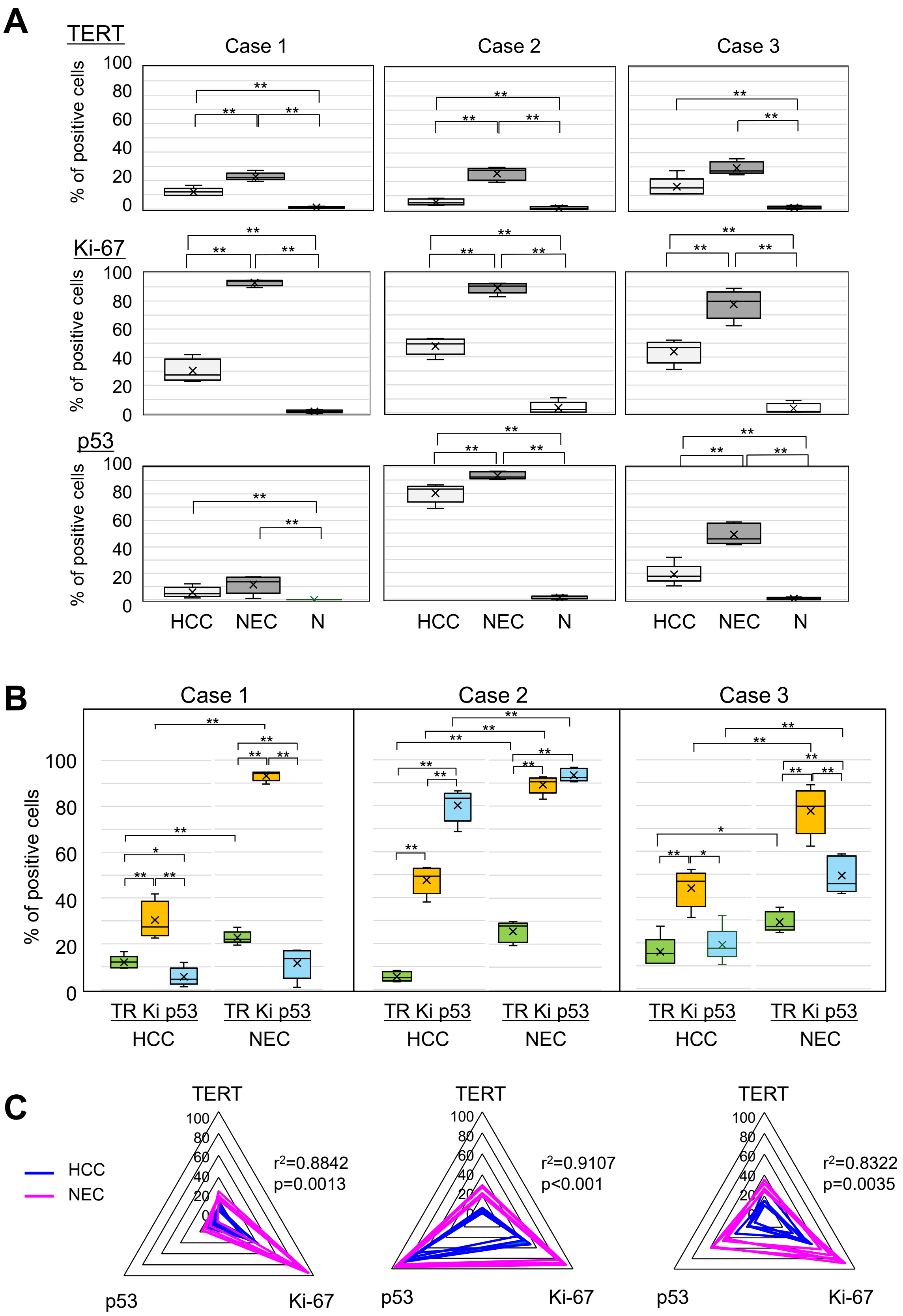

Figure S2. Maximum percentage of cells positive for TERT, Ki-67 and p53 proteins in the HCC and NEC components and nontumor liver regions (N). (A) Comparison of the positivity rates among the three components in each case. The values were obtained from five fields of view for each component with a relatively high frequency of each marker and were statistically analyzed by the Mann–Whitney U test (**, $p < 0.01$). (B) Comparisons of the maximum positivity rate among the three markers in each cancer component were statistically analyzed by the Mann–Whitney U test (**, $p < 0.01$ and *, $p < 0.05$). (C) Radar charts of the positivity rate of three markers in five fields of view for each cancer component: HCC (blue) and NEC (magenta). Linear regression of the positivity rate of three markers between the two cancer components was analyzed by ordinary least squares estimation.

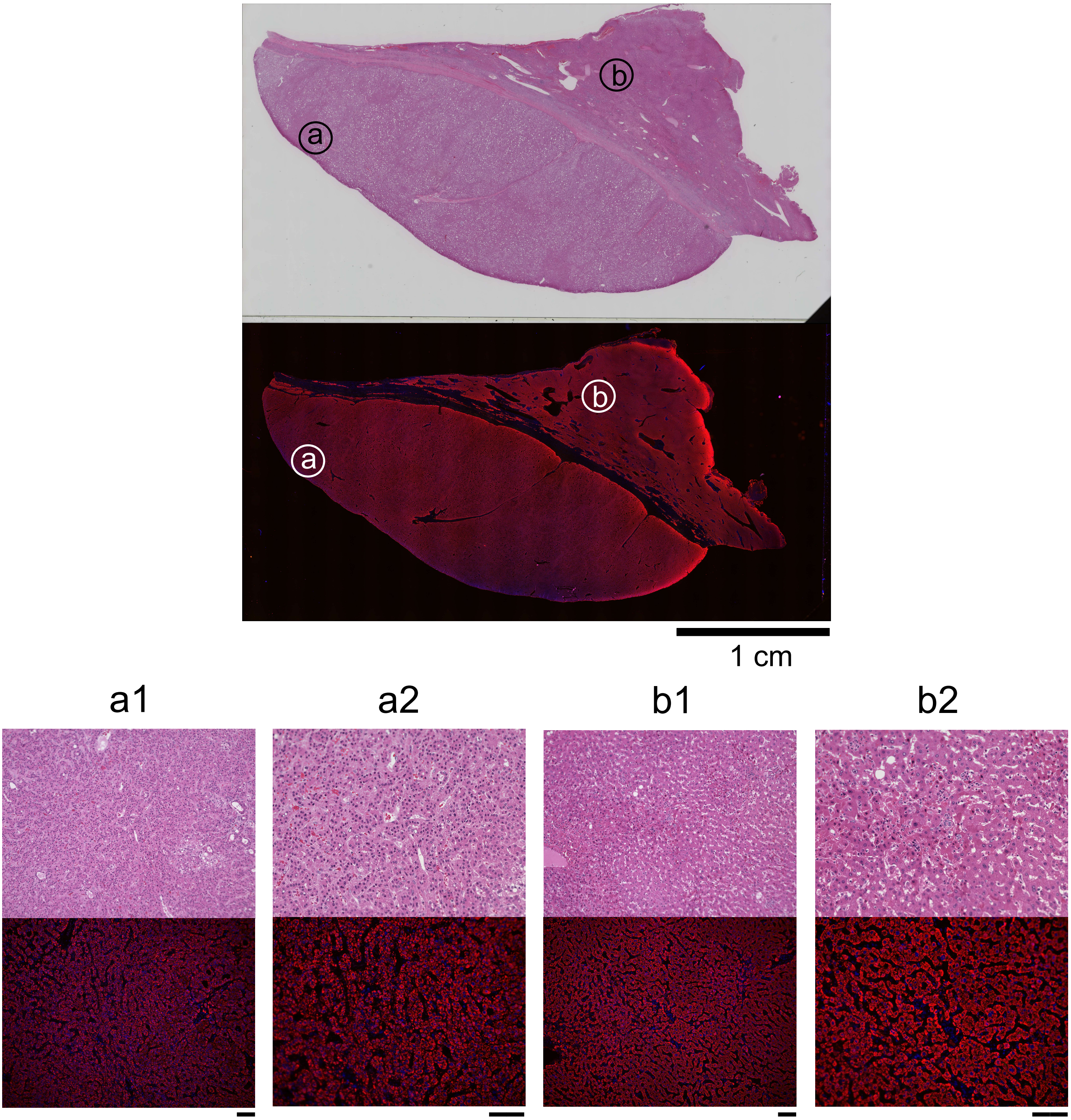

Figure S3. Double immunofluorescence (IF) for HepPar1 and INSM1 in the control HCC sample from a patient with HCC. Serial thin sections were stained with H&E (upper) and double IF (lower); whole images are shown. Microscopically magnified images of representative areas (a and b) are also shown below. a, HCC; b, nontumorous region. Images of a2 and b2 are magnified images of a1 and b1, respectively. Bars, 100 μm.

HepPar1 was homogenously stained in the whole parenchymal liver tissue sample and in the cytoplasm of HCC cells (a1, a2) and nontumorous hepatocytes (b1, b2). INSM1 was not detected in whole liver tissues.

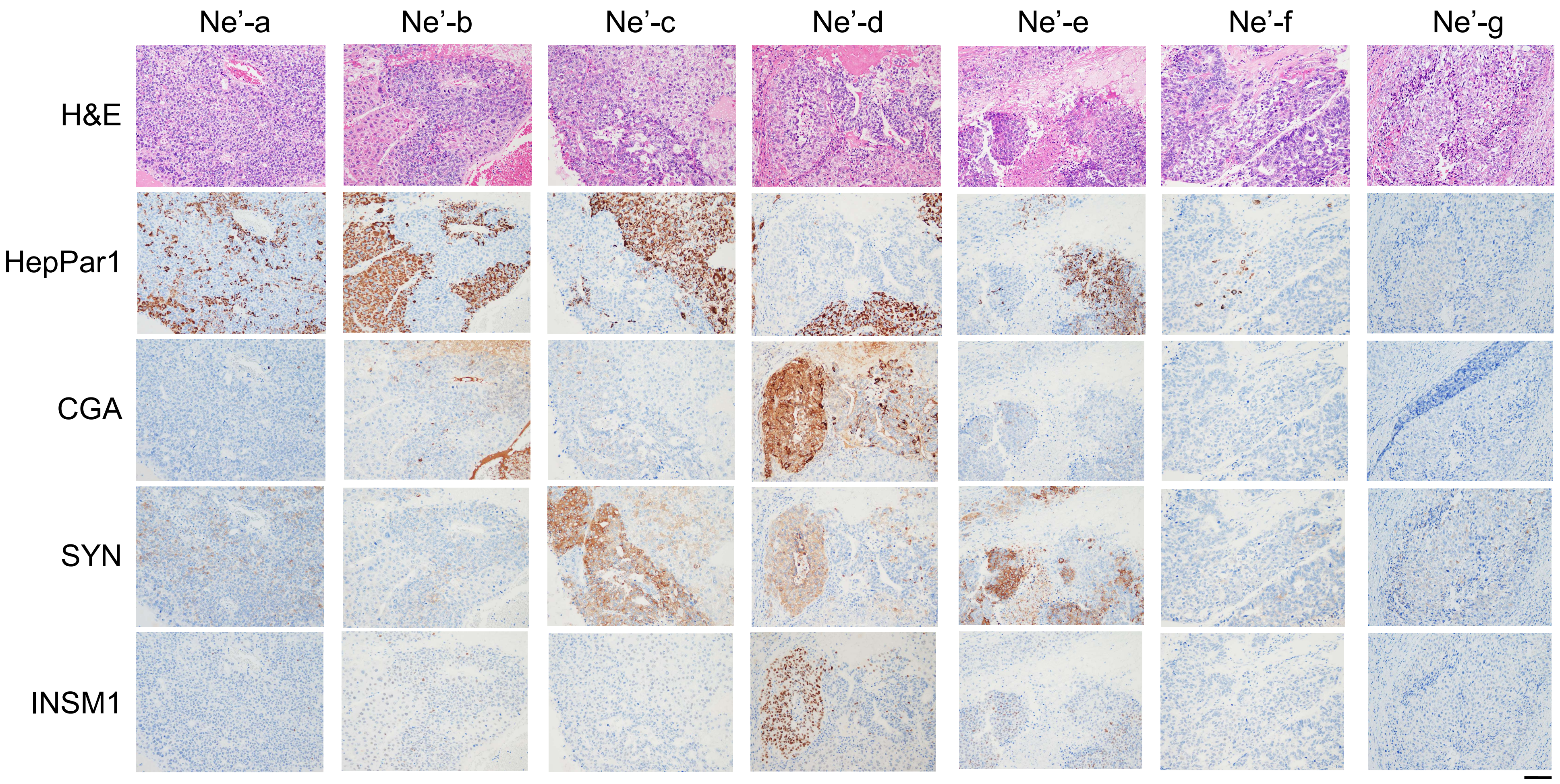

Figure S4. Images of NEC-like cells (Ne') subjected to immunohistochemical staining for HepPar1, CGA, SYN and INSM1 together with H&E staining in the tissue sample from case 3. Ne'-a to Ne'-f, NEC-like cells; Ne'-g, ambiguous NEC or NEC-like cells observed in an indeterminate NEC-like component (Figure 3Cd). Bars, 100 μm. Magnified images of H&E staining are shown in Figure 4D.

Ne'-a, patchy positive for HepPar1 and weakly positive for SYN; Ne'-b, negative for HepPar1 and very weakly positive for three NEC markers adjacent to HCC; Ne'-c, negative for HepPar1 and positive for SYN adjacent to HCC; Ne'-d, negative for HepPar1 and strongly positive for three NEC markers on the left but weakly positive for three NEC markers on the right; Ne'-e, negative for HepPar1 and positive for SYN and INSM1 on the left but positive for HepPar1 and partially positive for SYN and INSM1 on the right; Ne'-f, negative for HepPar1 and very weakly positive for SYN; Ne'-g, negative for HepPar1 and very weakly positive for SYN.

Table S2. Primary antibodies used for IHC

Antigen	Mouse mAb clone	Code	Dilution	Antigen retrieval*	Company
CGA	DAK-A3	M0869	1:200	pH 6.0/105°C,10 min	Agilent Technologies CA, USA
SYN	Rabbit polyclonal	M0010	1:50		
CD56	123C3	M7304	1:50		
HepPar1	OCH1E5	M7158	1:100	pH 9.0/105°C, 10 min	
TERT	A-6	sc-393013	1:50	pH 9.0/100°C, 20 min	Santa Cruz Biotechnology TX, USA
INSM1	A-8	sc-271408	1:100	pH 9.0/ 95°C-99°C, 30 min	
Ki-67	MIB-1	M7240	1:50		Agilent Technologies CA, USA
p53	DO-7	M7001	1:50		

*pH 6.0, Instant Buffer Citric Acid Buffer Solution, [AXEL] AS ONE, Osaka, Japan;
pH 9.0, EnVision FLEX Target Retrieval Solution, Agilent Technologies.
